# Animal Welfare and Resistance to Disease: Interaction of Affective States and the Immune System

**DOI:** 10.3389/fvets.2022.929805

**Published:** 2022-06-14

**Authors:** Sandra Düpjan, Marian Stamp Dawkins

**Affiliations:** ^1^Institute of Behavioural Physiology, Research Institute for Farm Animal Biology (FBN), Dummerstorf, Germany; ^2^Department of Zoology, University of Oxford, Oxford, United Kingdom

**Keywords:** affective state, immunity, welfare, gut microbiome, wellbeing, antibiotic resistance

## Abstract

Good management and improved standards of animal welfare are discussed as important ways of reducing the risk of infection in farm animals without medication. Increasing evidence from both humans and animals suggests that environments that promote wellbeing over stress and positive over negative emotions can reduce susceptibility to disease and/or lead to milder symptoms. We point out, however, that the relationship between welfare, immunity, and disease is highly complex and we caution against claiming more than the current evidence shows. The accumulating but sometimes equivocal evidence of close links between the brain, the gut microbiome, immunity, and welfare are discussed in the context of the known links between mental and physical health in humans. This evidence not only provides empirical support for the importance of good welfare as preventative medicine in animals but also indicates a variety of mechanisms by which good welfare can directly influence disease resistance. Finally, we outline what still needs to be done to explore the potential preventative effects of good welfare.

## Introduction

The spread of anti-microbial resistance (1, 2) and the devastating effects of diseases, such as influenza, covid, malaria, and TB, are grim reminders that even with the full resources of modern medicine at our disposal, we are only just keeping ahead in the arms race against current and emerging diseases. Furthermore, the current emphasis on the need to reduce the use of antibiotics e.g., (3, 4) removes an important means of safeguarding both human and animal health (5). There is thus an urgent need to find new ways of fighting disease, preferably ones that do not use medication.

In this study, we focus on the growing evidence that an important way of reducing the risk of infection may be through good management and improved standards of animal welfare. We draw on evidence from both humans and animals that environments that promote wellbeing over stress and positive over negative emotions can reduce susceptibility to disease or at least lead to milder symptoms and quicker recovery. However, the relationship between welfare, immunity, and disease is highly complex (6, 7), and there is no simple connection between “happiness” and resistance to infection. Therefore, we caution against claiming more than the evidence shows and outline what still needs to be done to explore the potential preventative effects of good welfare.

## Positive and Negative Wellbeing

Historically, the majority of studies on wellbeing, affective states, and health have focused on negative wellbeing, such as the negative effects of acute or chronic distress on morbidity and mortality (8–10). However, human health has long been acknowledged to be more than just the absence of disease (11). Similarly, animal welfare is not just the absence of stress and negative states (12, 13). Approaches such as the Five Freedoms (14) and Welfare Quality (15) emphasize the importance of going beyond physical health and including mental health as well. Physical health is ensured by keeping animals in clean, safe, and comfortable conditions and making sure that they have adequate access to water and nutritious food. Mental health is achieved by keeping them in conditions in which they have predominantly positive emotions associated with having what they like and want (16, 17).

## Wellbeing and Health—Evidence in Humans

In human medicine, the relatively new interdisciplinary field of Affective Immunology studies the links between emotion and the immune system. This covers both the way the immune system affects the emotional state and also the way that emotions alter the status of the immune status (7, 18). Studies investigating these links in humans use different approaches and constructs, making it difficult to interpret results and draw conclusions for non-human species. The term “wellbeing” includes eudaimonic wellbeing (whether someone sees their potential fulfilled or has a sense of purpose in life), hedonic wellbeing (having pleasurable experiences), and optimism (the expectation of positive results) (12, 13, 19). Health outcomes, on the other hand, are conceptualized as morbidity/recovery from disease, mortality/longevity, activation of certain parts of the immune system or associated systems (especially the cardiovascular system), or self-reported health. This diversity of concepts and measures, together with variation in sample sizes and potentially confounding variables (20), has led to controversial results and confusion about the direction of causation. However, systematic reviews and meta-analyses have helped to clarify the picture.

The meta-analyses by Chida and Steptoe (9) provides evidence for the protective effects of psychological wellbeing on mortality, although they are more controversial for already diseased populations (8). A more recent meta-analysis by DuPont et al. (13) found that hedonic wellbeing is linked to better hemodynamic recovery after stress, which might reduce the risk of developing stress-related cardiovascular diseases. Furthermore, good immune function is closely related to peoples' subjective reports of being happy and satisfied with their lives (21, 22). Conversely, impaired immune function has been found in people distressed by circumstances such as homelessness (23), and mental illnesses such as schizophrenia and depression are associated with an increase in the cellular immune response (24, 25) and neuronal cell surface antibodies (26, 27). Chronic stress can result in glucocorticoid receptor resistance that in turn leads to an inflammatory immune response that is pathologically out of control (28). On the other hand, conscientiousness has been linked to better health and more supportive social relationships (29), Tai Chi exercises can improve mental and physical health in persons with cardiovascular disease (30), and mindfulness-based training can improve emotional wellbeing as well as physical function and health (31). However, overall optimism does not seem to be linked to health as clearly as hedonic wellbeing (13) or not at all when controlling for other influencing factors in the statistical models (29). Even though optimistic patients might be more likely to persevere with therapy (29), optimistic judgements about health status might prevent someone from seeking timely medical advice (8). Indeed, Luo et al. (32) found that, during the COVID-19 pandemic, people worrying less about the disease showed less safety-seeking behavior, while perceived risk correlated negatively with wellbeing.

## Wellbeing and Health—Evidence in Animals

What is true for humans is now increasingly seen as applying to animals too (33). Human depression is associated both with chronic inflammation and compensatory responses to combat inflammation (34, 35), and there are clear parallels to stress responses in animals (36). For example, mice that are repeatedly subjected to stress such as being defeated in social encounters show an inflammation response throughout the body including enhanced neutrophil and cytokine activity (37). Social stress in pigs caused by fighting suppressed the immune response to a viral vaccine (38) while groups with low aggression social support can buffer acute stress responses in both humans (39) and other species (40, 41), with positive effects on the immune system (42, 43). It follows that providing stable social groups is a promising way of not only reducing injuries but also avoiding inflammation resulting from the stress of aggression. Giving animals the opportunity to feed undisturbed by conspecifics can have beneficial effects. In a cognitive enrichment experiment pigs had to learn their names and were then called to a feeding station, where they could then eat by themselves, and this had positive effects on health (44) and affective state (45–47).

The physical environment can also affect immune responses (48, 49). For example, enriching the environment of turkeys with “turkey trees” led to an increase in circulating white blood cells (50), and providing pigs with enrichments such as straw and branches resulted in a series of immunological changes including a higher percentage of T cells (51). Providing pigs with straw bedding can reduce the risk of gastric lesions (52), and young pigs with social and environmental enrichment were less susceptible to co-infection of PRRSV and *Actinobacillus pleuropneumoniae* and showed healthier lungs (53). Environmental enrichment early in life can also have positive effects on the development of the immune system and the establishment of gut microbiota in pigs (51).

## Wellbeing and Health—What We Still Need to Know

Although animal welfare as a way of controlling a disease is an attractive proposal with worldwide implications for both animal and human health, it is based on many ideas that are still largely untested (16, 33). The interactions between the brain, gut microbiome, and immune system are highly complex (36, 54, 55), and there is consequently no simple relationship between measures of immune activity and welfare. Evidence that improved animal welfare can lead to a reduction in infection may be true in some cases, but it is important not to claim more than the evidence shows.

One reason for caution is the complexity of the immune system itself. The vertebrate immune system consists of an extraordinary range of defense mechanisms, including the physical barrier of the skin that helps to prevent pathogens from entering the body as well as a whole range of specialized cells in the blood and lymphatic systems for detecting and destroying pathogens if they do get inside the body. In addition, an ecosystem of bacteria and other organisms living in the gut also has a profound effect on health in general and immune function in particular (54–56).

Immune responses occur in two stages which have very different implications for welfare. The innate or non-specific cellular immune system provides the first set of responses to infection or injury including the production of bacteria-destroying granulocytes, the release of cytokines, and local inflammation together with a whole range of sickness responses such as fever. It is an all-purpose emergency reaction, stimulated by a wide range of dangers and involving many different parts of the body. It needs such a high level of nutrients to keep it functioning that fighting disease may result in more resources being put into immune function and less into growth (57). Conversely, when animals become stressed, a cascade of hormonal responses including the release of corticosteroids or stress hormones shifts the entire metabolism away from immune responses and toward releasing readily available energy for taking some kind of action.

The second stage in the immune response is the more targeted “acquired” immunity stage which consists of the development of specific antibodies against particular diseases, in which the body “discovers” the correct antibody against a particular disease and then clones multiple copies. A relatively small number of specific antigens then provide long-lasting protection against infection.

Given the complexity of these immune reactions and their interactions with both the gut microbiome and the emotions, there are also many different ways in which immunity can affect and be affected by emotional state (10, 58). First, changes in the immune system, such as inflammation, may directly affect, and be affected by, the emotional state (7, 13, 59). Second, immunity and emotional state may be linked by more indirect routes, for example, via effects on the cardiovascular system (e.g., (8, 13, 60) and the gut microbiome (61). The gut microbiome is a complex community of viruses, bacteria, archaea, and eukaryotes, the composition of which is strongly influenced by factors such as diet and the neurological and endocrinological responses of the body to stress (62). In turn, the microbiome affects how the body responds to stress and to disease challenges (61, 63, 64). Even more indirectly, the immune response can be influenced by behavioral changes such as dietary choice, rest or activity, and avoidance of other individuals, all of which can result in a reduced risk of disease and/or faster recovery from disease. Looking through the literature on the links between wellbeing and health in humans, despite known physiological pathways (8, 10), the most meaningful pathway between immunity and health seems to be via behavior (20). The happiest and most conscientious individuals tend to make less risky decisions and instead engage in behaviors that improve their health, such as a healthier diet and regular exercise (9, 29).

There are thus many different ways in which improving standards of animal welfare might influence immunity because there are so many different ways in which the immune system is influenced by, and exerts influence on, so many other systems of the body. There is much that we still do not understand and much we still have to learn. It is also important to remember that, even if a particular practice, such as an improvement in welfare management, affects immune responses, this is only the first step toward the much stronger claim that improved welfare protects against disease.

Many studies on the effects of welfare on disease, including most of those cited in this article, have been conducted by comparing the body's immune response in different conditions and then drawing conclusions about the potential effect this might have on the ability to resist actual infection. From such evidence, it is often concluded that keeping animals in the higher welfare conditions would improve their ability to resist disease. Now while this is a plausible inference from the evidence presented, it is by no means certain that this would be the case out there in the real world. A disease may be so severe that the immune system, although making a valiant attempt to protect, will be ineffective at resisting infection.

While tests of immune response under controlled conditions are an essential preliminary, we also need on-farm studies to demonstrate that farm animals can actually realize the full potential of their immune function under real-world conditions. The ultimate test of the protective effect of good animal welfare must therefore be evidence that, under commercial farm conditions, animals kept in high welfare conditions are less likely to fall victim to disease or more likely to recover quickly, along with guidance about the limitations of what improved welfare can achieve. To exaggerate the effects of good management on disease resistance could be as counter-productive as ignoring the effects of good welfare altogether. There is an urgent need for research in this area and it needs to be based on evidence collected in the real world.

## The Path to Better Welfare

Even knowing more about the relationship between disease resistance and welfare will not, however, resolve fundamental issues about how to implement them in practice. Indeed, there may be conflicts about how best to reduce the risks of different diseases. In dairy cattle, access to pasture can reduce the risk of mastitis, claw health, and other health issues, but this can come with a higher risk for parasitism and malnutrition (65).

For some people “improving welfare” means moving toward free-range systems and away from intensive indoor methods of production altogether, despite these extra risks. There is a widespread assumption that animals are more likely to be healthy and to have positive emotions if they can show more “natural behaviour” (66–68) and the health risk of reduced biosecurity is judged as less important than the positive welfare benefits of a more “natural” life (69). In complete contrast, other people see the route to better welfare being through the increased use of technology that allows not only improved biosecurity but the provision of optimal environmental conditions that allow the immune system to function more effectively. For example, heat stress is a major form of poor welfare, leading to a variety of pathologies, including making animals more susceptible to infection (51, 70). Amongst other effects, heat stress damages the intestinal mucosa of poultry, making it more likely that endotoxins and even bacteria will enter the bloodstream (71). The controlled indoor conditions achievable by smart farming can do a great deal to reduce heat and other stressors (72). On the other hand, there may be adverse consequences for resistance to other diseases caused by high stocking densities or other features of intensive systems (73).

These two opposite views of how to improve welfare—more extensive outside “natural” living versus more intensive indoor technology-led living—clearly have very different implications for disease risk, both for the chances of animals encountering infective organisms in the first place and also for how their bodies might later react to being infected. There are no simple answers and future developments will need to find a balance between the costs and benefits of different systems. Animal welfare is only one of many weapons we have in the fight against infection, one that has perhaps not yet been fully appreciated but one where our knowledge is still very incomplete.

## Conclusions

The hypothesis that good animal welfare optimizes the conditions in which the body's own natural defenses operate most effectively and can therefore be an effective weapon against infectious disease is a potential of major significance to both animal and human health. However, it currently lacks good supporting evidence, and it is important not to oversell the idea or exaggerate the ability of good animal welfare to substitute for medication. To test the hypothesis, it will be necessary to demonstrate that high welfare conditions (carefully defined) actually do protect against disease, not just in theory, in the lab, or in experimental conditions but in real-world commercial conditions. There may have to be many caveats, such as that good welfare can offer protection with some diseases but not others or that some aspects of “good welfare”, such as avoiding diseases associated with overheating, may be in conflict with what is meant by “good welfare” in some other respect such as allowing animals to range outdoors. With two things as complex as disease prevention and animal welfare, we should not expect simple solutions.

However, the accumulating evidence of close links between the brain, the gut microbiome, immunity, and welfare as well as the known links between mental and physical health in humans not only provides empirical support for the importance of good welfare as preventative medicine but also indicates a variety of mechanisms by which good welfare can directly influence disease resistance.

## Data Availability Statement

The original contributions presented in the study are included in the article/supplementary material, further inquiries can be directed to the corresponding author.

## Author Contributions

All authors listed have made a substantial, direct, and intellectual contribution to the work and approved it for publication.

## Funding

The publication of this article was funded by the Open Access Fund of the FBN.

## Conflict of Interest

The authors declare that the research was conducted in the absence of any commercial or financial relationships that could be construed as a potential conflict of interest.

## Publisher's Note

All claims expressed in this article are solely those of the authors and do not necessarily represent those of their affiliated organizations, or those of the publisher, the editors and the reviewers. Any product that may be evaluated in this article, or claim that may be made by its manufacturer, is not guaranteed or endorsed by the publisher.

## References

[1] Hudson JA Frewer LJ Jones G Brereton PA Whittingham MJ Stewart G. The agri-food chain and antimicrobial resistance: A review. Trends Food Sci Technol. (2017) 69:131–47. 10.1016/j.tifs.2017.09.007

[2] Aidara-Kane A Angulo FJ Conly JM Minato Y Silbergeld EK McEwen SA. for the WHO Guideline Development Group. World health Organisation (WHO) guidelines on use of medically important microbials in food-processing animals. Antimicrob Resist Infect Control. (2018) 7:7. 10.1186/s13756-017-0294-9 29375825 PMC5772708

[3] ECDC (European Centre for Disease Prevention and Control) EFSA (European Food Safety Authority) and EMA (European Medicines Agency). 2017 ECDC/EFSA/EMA second joint report on the integrated analysis of the consumption of antimicrobial agents and occurrence of antimicrobial resistance in bacteria from humans and food-producing animals – Joint Interagency Antimicrobial Consumption and Resistance Analysis (JIACRA) Report. EFSA J. (2017) 15:135. 10.2903/j.efsa.2017.4872 32625542 PMC7009874

[4] Ying GG He LY Ying AJ Zhang QQ Liu YS Zhao JI. China must reduce its antibiotic use. Environ Sci Technol. (2017) 51:1072–3. 10.1021/acs.est.6b06424 28094517

[5] Rojo-Gimeno C Postma M Dewulf J Hogeveen H Lauwers L Wauters E. Farm-economic analysis of reducing antimicrobial use while adopting improved management strategies on farrow-to-finish pig farms. Prev Vet Med. (2016) 129:74–87. 10.1016/j.prevetmed.2016.05.001 27317325

[6] Berghman LR. Immune responses to improving welfare. Poult Sci. (2016) 95:2216–8. 10.3382/ps/pew159 27143775 PMC4983685

[7] D'Acquisto F. Affective immunology: Where emotions and the immune response converge. Dialogues Clin Neurosci. (2017) 19:9–19. 10.31887/DCNS.2017.19.1/fdacquisto 28566943 PMC5442367

[8] Pressman SD Cohen S. Does positive affect influence health? Psychol Bull. (2005) 131:925–71. 10.1037/0033-2909.131.6.925 16351329

[9] Chida Y Steptoe A. Positive psychological wellbeing and mortality: A quantitative review of prospective observational studies. Psychosom Med. (2008) 70:741–56. 10.1097/PSY.0b013e31818105ba 18725425

[10] Huffman JC Legler SR Boehm JK. Positive psychological wellbeing and health in patients with heart disease: A brief review. Future Cardiol. (2017) 13:443–50. 10.2217/fca-2017-0016 28828901

[11] World Health Organization. Preamble to the Constitution of the World Health Organization. In: Official records of the World Health Organization. Geneva: World Health Organization. (1948) p. 100.

[12] Ryff CD Singer BH Love GD. Positive health: Connecting wellbeing with biology. Philos Trans R Soc Lond B. (2004) 359:1383–94. 10.1098/rstb.2004.1521 15347530 PMC1693417

[13] DuPont CM Weis TM Manuck SB Marsland AL Matthews KA Gianaros PJ. Does wellbeing associate with stress physiology? A systematic review and meta-analysis. Health Psychol. (2020) 39:879–90. 10.1037/hea0000979 32686951

[14] FAWC (Farm Animal Welfare Council). Farm Animal Welfare in Great Britain: Past, Present Future. London: FAWC (2009). Available online at: https://assets.publishing.service.gov.uk/government/uploads/system/uploads/attachment_data/file/319292/Farm_Animal_Welfare_in_Great_Britain_-_Past__Present_and_Future.pdf

[15] Welfare Quality®. Welfare Quality® Assessment Protocol for Poultry (Broiler, Laying Hens). Lelystad, The Netherlands: Welfare Quality Consortium (2009).

[16] Boissy A Manteuffel G Jensen MB Moe RO Spruijt B Keeling LJ. Assessment of positive emotions in animals to improve their welfare. Physiol Behav. (2007) 92:375–97. 10.1016/j.physbeh.2007.02.003 17428510

[17] Green TC Mellor DJ. Extending ideas about animal welfare assessment to include ‘quality of life' and related concepts. N. Zealand Vet J. (2011) 59:263–71. 10.1080/00480169.2011.610283 22040330

[18] Vasile C. Mental health and immunity (Review). Exp Ther Med. (2020) 20:211. 10.3892/etm.2020.9341 33149775 PMC7604758

[19] Cohen R Bavishi C Rozanski A. Purpose in life and its relationship to all-cause mortality and cardiovascular events: a meta-analysis. Psychosom Med. (2016) 78:122–33. 10.1097/PSY.0000000000000274 26630073

[20] Steptoe A. Happiness and health. Annu Rev Public Health. (2019) 40:339–59. 10.1146/annurev-publhealth-040218-044150 30601719

[21] Nakata A Takahashi M Irie M Swanson NG. Job satisfaction is associated with elevated natural killer cell immunity among healthy, white-collar employees. Brain, Behavior and Immunity. (2010) 24:1268–75. 10.1016/j.bbi.2010.05.004 20561922

[22] Takao Y Okuno Y Mori Y Asasa H Yamanishi K Iso H. Associations of perceived mental stress, sense of purpose in life, and negative life events with the risk of incident herpes zoster and postherpetic neuralgia: The SHEZ study. Am J Epidemiol. (2018) 187:251–9. 10.1093/aje/kwx249 29036443

[23] Arranz L de Vicente A Muñoz M De la Fuente M. Impaired immune function in a homeless population with stress-related disorders. Neuroimmunomodulation. (2009) 16:251–60. 10.1159/000212386 19365149

[24] Maes M. Depression as an inflammatory disease, but cell-mediated immune activation is the key component of depression. Prog Neuropsychopharmacol Biol Psychiatry. (2011) 35:664–75. 10.1016/j.pnpbp.2010.06.014 20599581

[25] Horsdal HT Köhler-Forsberg O Benros ME Gasse C. C-reactive protein and white blood cell levels in schizophrenia, bipolar disorders and depression-associations with mortality and psychiatric outcomes: a population-based study. Eur Psychiatry. (2017) 44:164–72. 10.1016/j.eurpsy.2017.04.012 28645055

[26] Steiner J Bogerts B Sarnyai Z Walter M Gos T Bernstein HG. Bridging the gap between the immune and glutamate hypotheses of schizophrenia and major depression: Potential role of glial NMDA receptor modulators and impaired blood-brain barrier integrity. World J Biol Psychiatry. (2012) 13:482–92. 10.3109/15622975.2011.583941 21707463

[27] Lennox BR Palmer-Cooper EC Pollak T Hainsworth J Marks J Jacobson L. PPiP study team. Prevalence and clinical characteristics of serum neuronal cell surface antibodies in first-episode psychosis: a case-control study. Lancet Psychiatry. (2017) 4:42–8. 10.1016/S2215-0366(16)30375-3 27965002 PMC5890880

[28] Cohen S Janicki-Deverts D Doyle WJ Miller GE Frank E Rabin BS. Chronic stress, glucocorticoid receptor resistance, inflammation, and disease risk. PNAS. (2012) 109:5995–9. 10.1073/pnas.1118355109 22474371 PMC3341031

[29] Friedman HS Kern ML. Personality, wellbeing, and health. Annu Rev Psychol. (2014) 65:719–42. 10.1146/annurev-psych-010213-115123 24405364

[30] Taylor-Piliae RE Finley BA. Tai Chi exercise for psychological wellbeing among adults with cardiovascular disease: A systematic review and meta-analysis. Eur J Cardiovasc Nurs. (2020) 19:580–91. 10.1177/1474515120926068 32515204

[31] Zhang J Xu R Wang B Wang J. Effects of mindfulness-based therapy for patients with breast cancer: a systematic review and meta-analysis. Complement Ther Med. (2016) 26:1–10. 10.1016/j.ctim.2016.02.012 27261975

[32] Luo YF Shen HY Yang SC Chen LC. The relationships among anxiety, subjective wellbeing, media consumption, and safety-seeking behaviors during the COVID-19 epidemic. Int J Environ Res Public Health. (2021) 18:13189. 10.3390/ijerph182413189 34948796 PMC8700923

[33] Dawkins MS. Animal welfare as preventative medicine. Animal Welfare. (2019) 28:137–41. 10.7120/09627286.28.2.137

[34] Berk M Williams LJ Jacka FN O'Neil A Pasco JA Moylan S. So depression is an inflammatory disease, but where does the inflammation come from? BMC Med. (2013) 11:200. 10.1186/1741-7015-11-200 24228900 PMC3846682

[35] Talarowska ME Kowalczyk M Maes M Carvalho A Su KP Szemraj J. Immune to happiness-inflammatory process indicators and depressive personality traits. Archives of Medical Science. (2020) 16:848–57. 10.5114/aoms.2019.83146 32542087 PMC7286335

[36] Dantzer R O'Connor JC Freund GG Johnson RW Kelley KW. From inflammation to sickness and depression: when the immune system subjugates the brain. Nat Rev Neurosci. (2008) 9:46–57. https://dx.doi.org/10.1038%2Fnrn2297 10.1038/nrn2297 18073775 PMC2919277

[37] Lafuse WP Gearinger R Fisher S Nealer C Mackos AR Bailey MT. Exposure to a social stressor induces translocation of commensal *Lactobacilli* to the spleen and priming of the innate immune system. J Immunol. (2017)198:2383–93. 10.4049/jimmunol.1601269 28167628 PMC5340647

[38] de Groot J Ruis MAW Scholten JW Koolhaas JM Boersma WJA. Long-term effects of social stress on antiviral immunity in pigs. Physiol Behav. (2001) 73:145–58. 10.1016/S0031-9384(01)00472-3 11399306

[39] Cohen S Wills TA. Stress, social support, and the buffering hypothesis. Psychological Bull. (1985) 98:310–57. 10.1037/0033-2909.98.2.310 3901065

[40] Kikusui T Winslow JT Mori Y. Social buffering: Relief from stress and anxiety. Philos Trans R Soc Lond, B. (2006) 361:2215–28. 10.1098/rstb.2006.1941 17118934 PMC1764848

[41] Kanitz E Hameister T Tuchscherer M Tuchscherer A Puppe B. Social support attenuates the adverse consequences of social deprivation stress in domestic piglets. Horm Behav. (2014) 65:203–10. 10.1016/j.yhbeh.2014.01.007 24486118

[42] Tuchscherer M Kanitz E Puppe B Hameister T Tuchscherer A. Social support modulates splenocyte glucocorticoid sensitivity in piglets exposed to social deprivation stress. Physiol Behav. (2014) 131:25–32. 10.1016/j.physbeh.2014.04.010 24732413

[43] Tuchscherer M Kanitz E Tuchscherer A Puppe B. Effects of social support on glucocorticoid sensitivity of lymphocytes in socially deprived piglets. Stress. (2016) 19:325–32. 10.1080/10253890.2016.1179276 27160343

[44] Ernst K Tuchscherer M Kanitz E Puppe B Manteuffel G. Effects of attention and rewarded activity on immune parameters and wound healing in pigs. Physiol Behav. (2006) 89:448–56. 10.1016/j.physbeh.2006.07.001 16904140

[45] Puppe B Ernst K Schön PC Manteuffel G. Cognitive enrichment affects behavioural reactivity in domestic pigs. Appl Anim Behav Sci. (2007) 105:75–86. 10.1016/j.applanim.2006.05.016

[46] Kalbe C Puppe B. Long-term cognitive enrichment affects opioid receptor expression in the amygdala of domestic pigs. Genes Brain and Behavior. (2010) 9:75–83. 10.1111/j.1601-183X.2009.00536.x 19804558

[47] Zebunke M Langbein J Manteuffel G Puppe B. Autonomic reactions indicating positive affect during acoustic reward learning in domestic pigs. Anim Behav. (2011) 81:481–9. 10.1016/j.anbehav.2010.11.023

[48] Brod S Gobbetti T Gittens B Ono M Perretti M D'Acquisto F. The impact of environmental enrichment on the murine inflammatory immune response. JCI Insight. 2 (2017) e90723. 10.1172/jci.insight.90723 28405616 PMC5374068

[49] Luo L Jansen CA Bolhuis JE Arts JAJ Kemp B Parmentier HK. Early and later life environmental enrichment affect specific antibody responses and blood leukocyte subpopulations in pigs. Physiol Behav. (2020) 217:112799. 10.1016/j.physbeh.2020.112799 31923451

[50] Lindenwald R Schuberth HJ Spindler B Rautenschlein S. Influence of environmental enrichment on circulating white blood cell counts and behaviour of females turkeys. Poult Sci. (2021) 100:101360. 10.1016/j.psj.2021.101360 34320453 PMC8327346

[51] Wen C van Dixhoorn I Schokker D Woelders H Stockhofe-Zurwieden N Rebel JMJ. Environmentally enriched housing conditions affect pig welfare, immune system and gut microbiota in early life. Animal Microbiome. (2021) 3:52. 10.1186/s42523-021-00115-2 34321110 PMC8320228

[52] Bolhuis JE van den Brand H Staals S Gerrits WJJ. Effects of pregelatinized vs native potato starch on intestinal weight and stomach lesions of pigs housed in barren pens or on straw bedding. Livestock Science. (2007) 109:108–10. 10.1016/j.livsci.2007.01.100

[53] van Dixhoorn IDE Reimert I Middelkoop J Bolhuis JE Wisselink HJ Groot Koerkamp PWG. Enriched housing reduces disease susceptibility to co-infection with porcine reproductive and respiratory virus (PRRSV) and *Actinobacillus pleuropneumoniae* (*A. Pleuropneumoniae)* in young pigs. PLoS ONE. (2016) 11:e0161832. 10.1371/journal.pone.0161832 27606818 PMC5015855

[54] Bailey MT Cryan JF. The microbiome as a key regulator of brain, behaviour and immunity: Commentary on the 2017 named series. Brain, Behavior, and Immunity. (2017) 66:18–22. 10.1016/j.bbi.2017.08.017 28843452

[55] Leonard BE. Inflammation and depression: causal or coincidental link to the pathophysiology? Acta Neuropsychiatr. (2018) 30:1–16. 10.1017/neu.2016.69 28112061

[56] Yeoman CJ White BA. Gastrointestinal tract microbiota and probiotics in production animals. Annu Rev Anim Biosci. (2014) 2:469–86. 10.1146/annurev-animal-022513-114149 25384152

[57] Brock PM Hall AJ Goodman SJ Cruz M Acevedo-Whitehouse K. Immune activity. Body condition and human-associated environmental impacts in a wild marine mammal. PLoS ONE. (2013) 8:e67132. 10.1371/journal.pone.0067132 23840603 PMC3695956

[58] Sin NL. The protective role of positive wellbeing in cardiovascular disease: Review of current evidence, mechanisms, and clinical implications. Curr Cardiol Rep. (2016) 18:106. 10.1007/s11886-016-0792-z 27612475 PMC5060088

[59] Ironson G Banerjee N Fitch C Krause N. Positive emotional wellbeing, health behaviors, and inflammation measured by C-Reactive protein. Soc Sci Med. (2018) 197:235–43. 10.1016/j.socscimed.2017.06.020 28701268

[60] Chida Y Hamer M. Chronic psychosocial factors and acute physiological responses to laboratory-induced stress in healthy populations: a quantitative review of 30 years of investigations. Psychol Bull. (2008) 134:829–85. 10.1037/a0013342 18954159

[61] Kraimi N Dawkins M Gebhardt-Henrich Velge P Rychlik I Volf J. Influence of the microbiota-gut-brain axis on behaviour and welfare in farm animals: a review. Physiol Behav. (2019) 210:112658. 10.1016/j.physbeh.2019.112658 31430443

[62] Villageliu DN Lyte M. Microbial endocrinology: Why the intersection of microbiology and neurobiology matters to poultry health. Poult Sci. (2017) 96:2501–8. 10.3382/ps/pex148 29050443

[63] Dinan TG Cryan JF. Regulation of the stress response by the gut microbiota: Implications for psychoneuroendocrinology. Psychoneuroendocrinology. (2012) 37:1369–78. 10.1016/j.psyneuen.2012.03.007 22483040

[64] Brod S Rattazzi L Piras G D'Acquisto F. 'As above, so below' examining the interplay between emotion and the immune system. Immunology. (2014) 143:311–8. 10.1111/imm.12341 24943894 PMC4212945

[65] Mee JF Boyle LA. Assessing whether dairy cow welfare is “better” in pasture-based than in confinement-based management systems. N Z Vet J. (2020) 68:168–77. 10.1080/00480169.2020.1721034 31973680

[66] Rabin LA. Maintaining behavioural biodiversity in captivity for conservation: natural behaviour management. Animal Welfare. (2003) 12:85–94.

[67] Yeates JW. How good? Ethical criteria for a ‘Good Life' for farm animals. J Agric Environ Ethics. (2017) 30:23–35. 10.1007/s10806-017-9650-2

[68] Yeates JW. Naturalness and animal welfare. Animals. (2018) 8:53. 10.3390/ani8040053 29621140 PMC5946137

[69] Nussbaum MC. Beyond ‘compassion and humanity': Justice for non-human animals. In: Sunstein CS Nussbaum MC editors. Animal Rights: Current Debates and New Directions. Oxford: Oxford University Press. (2004). p. 299–320. 10.1093/acprof:oso/9780195305104.003.0015

[70] Bilal RM Hassan FU Farag MR Nasir TA Ragni M Mahgoub HAM Alagawany M. Thermal stress and high stocking densities in poultry farms: potential effects and mitigation strategies. J Therm Biol. (2021) 99:102944. 10.1016/j.jtherbio.2021.102944 34420608

[71] Alhenaky A Abdelqader A Abuajamieh M Al-Fataftah AR. The effect of heat stress on intestinal integrity and *Salmonella* invasion in broiler birds. J Therm Biol. (2017) 70:9–14. 10.1016/j.jtherbio.2017.10.015 29108563

[72] Dawkins MS. Does smart farming improve or damage animal welfare? Technology and what animals want. Front Anim Sci. (2021) 2:736536. 10.3389/fanim.2021.736536

[73] Wathes CM Kristensen HH Aerts JM Berckmans D. Is precision livestock farming an engineer's daydream or nightmare, an animal's friend or foe, and a farmer's panacea or pitfall? Comput Electron Agric. (2008) 64:2–10. 10.1016/j.compag.2008.05.005

